# Supplementary material for: Functional and diffusion MRI reveal the neurophysiological basis of neonates’ noxious-stimulus evoked brain activity
Source: Nat Commun. 2021 May 12;12:2744. doi: 10.1038/s41467-021-22960-0 (PMC8115252; doi:10.1038/s41467-021-22960-0)
Supplement: Supplementary file 1 — Supplementary Information [file 41467_2021_22960_MOESM1_ESM.pdf]

Supplementary Information

Functional and diffusion MRI reveal the neurophysiological basis of neonates' noxious-stimulus evoked brain activity

Baxter et al.

Supplementary Notes

Noxious-evoked response amplitudes and fMRI timeseries data quality

Noxious-evoked response HRF fit

Neonates' noxious-evoked response peristimulus timeseries data quality was assessed using the goodness-of-fit between a neonate's trial-averaged (10 trials) evoked response waveform and the haemodynamic response function (HRF) waveform. Voxels in each neonate's noxious-evoked response map were segmented into three classes using FSL's mixture modelling approach: null, positive BOLD (blood oxygen level dependent signal), and negative BOLD[1]. Peristimulus timeseries plots were constructed using the default method in FSL's FEAT, using the noxious-evoked response map voxel with largest z-statistic. Using the mixture modelling outputs, the voxel with largest positive z-statistic was extracted from the positive BOLD response class, and the voxel with largest negative z-statistic was extracted from the negative BOLD response class. This approach ensured the selected voxels do not come from regions lacking activation (i.e. the null class) but with a spurious good fit, and that the selected voxels are representative of the best HRF fit for that subject (i.e. the largest z-statistic). The peristimulus timeseries plots include all timepoints for all trials, the trial average response, and the HRF fit (Supplementary Figure 1). To assess goodness-of-fit between the data trial average response and the HRF fit, the Pearson correlation coefficient was calculated and converted to a z-score using the Fisher r-to-z transformation. Due to the scale invariance of the Pearson correlation coefficient, the correlation should indicate the quality of alignment between the HRF and the data and not capture amplitude effects.

Resting-state network timeseries outliers

Neonates' resting-state whole scan session timeseries data quality was assessed by quantifying the outlier timepoint content. The underlying BOLD fluctuations should follow slow and smooth fluctuations, so the number of outliers across networks may give an indication of how faithfully our network timeseries reflect these BOLD fluctuations. The presence of outliers in network timeseries typically indicate sudden changes in signal intensity, which can indicate head motion contamination. Outliers were independently detected for each network timeseries using the "quartiles method": outliers defined as values more than 1.5 interquartile ranges above the upper quartile or below the lower quartile (Supplementary Figure 2). For each neonate, a count of total number of outliers across all nine networks was made as well as the percentage of unique timepoints labelled an outlier in at least one network timeseries. Larger outlier counts and percentages were taken to indicate a poorer relationship between the observed network timeseries and the underlying BOLD timeseries. While the presence of these outliers will not strongly influence our measures of network amplitudes due to the use of median absolute deviation (which is relatively robust to outliers), the number of outliers was taken to indicate the quality of the network timeseries underpinning the estimated network amplitudes.

Noxious-evoked response amplitudes vs fMRI timeseries data quality

To assess the potential that individual variability in noxious-evoked response amplitudes may reflect variability in data timeseries quality, we assessed the association between neonates' noxious-evoked response amplitudes and the measures of data quality described above for the noxious-

evoked responses (HRF goodness-of-fit z-scores, Supplementary Figure 1) and resting-state network timeseries (total number of outlier timepoints and percentage of unique outlier timepoints, Supplementary Figure 2). The associations are displayed in Supplementary Figure 3.

In all cases, we performed Pearson correlation tests with statistical significance assessed in FSL's PALM[2] using a two-tailed permutation test with 10,000 permutations. We did not correct for multiple testing, and no tests displayed significance or trends towards significance in the uncorrected p-values. The Pearson correlation coefficients and associated p-values are displayed in Supplementary Figure 3. These results suggest that the observed variability in noxious-evoked response amplitudes (i) do not reflect noxious-evoked response data quality related to the degree of (mis)alignment when fitting the HRF, or (ii) do not correspond to resting-state network timeseries data quality, and by extension, the quality of the estimated resting-state network timeseries amplitudes.

Resting-state coupling: global effects, non-uniformity, and nociception-relevance
In our core analyses, we tested three sets of predictors for their capacity to predict neonates' noxious-evoked response amplitudes: nine resting-state networks, three resting-state imaging confounds, and six clinical variables. The multivariate resting-state network amplitudes could predict neonates' noxious-evoked response amplitudes with statistically significant accuracy (main text Table 2). The resting-state imaging confound multivariate model, which included head motion as well as CSF and white matter signal amplitudes, was not predictive, demonstrating that the resting-state network predictions could not be attributed to head motion or artefactual global signals. Finally, the multivariate clinical variables model (which included postmenstrual, gestational, and postnatal age, as well as birth weight, sex, and total brain volume) was not predictive, suggesting these biologically interesting variables similarly could not account for the resting-state network predictions. Here, we examine each of these 18 predictors individually to gain further insight into our prediction results.

Univariate correlation analysis
Performing univariate correlations between each individual resting-state network's amplitudes and the noxious-evoked response amplitudes (adjusted for resting-state and noxious-evoked response imaging confounds, see Methods), eight of nine resting-state network amplitudes exhibited positive correlations with noxious-evoked response amplitudes (Supplementary Figure 4a) – the executive control network had a negligible negative correlation coefficient ($r=-0.03$). This large proportion of positive associations suggests a near global effect that may be attributable to brain maturity, such that larger evoked response amplitudes and larger resting-state network amplitudes are associated with a more functionally mature brain (see Discussion in main text). Statistical significance for each correlation was assessed using permutation testing in FSL's PALM using 10,000 permutations and FWER-correction for multiple testing across the 18 univariate tests. Of the 18 variables, only two resting-state network correlations were statistically significant: somatomotor network (SMN; $r=0.73$, $p= 0.011$) and the occipital pole visual network (VNop; $r= 0.77$, $p=0.003$). Interestingly the role of visual brain regions in pain and nociception is also reported in the adult literature – see the recent adult pain prediction paper by Spisak and colleagues[3] and the discussion and further

reference therein. No individual resting-state imaging confound or clinical variable exhibited trends towards significance, even without corrections for multiple testing.

Univariate prediction analysis

Despite the trend towards a global positive association between all resting-state network amplitudes and noxious-evoked response amplitudes, the association strengths across networks were not uniform but varied largely from network to network (Supplementary Figure 4a). To identify individual networks most predictive of neonates' noxious-evoked response amplitudes and to further assess the network-to-network variability in univariate correlations strengths with noxious-evoked response amplitudes, we examined univariate predictions. For completeness, we performed this univariate prediction analysis for all 18 predictors. Each univariate prediction model was trained in an identical manner to our multivariate prediction models described in the main text, using a linear support vector regression model, leave-one-out cross validation, and grid search optimisation of regularisation strength. Each of the nine univariate resting-state network amplitude prediction models included cross-validated confound regression using the three resting-state imaging confounds to adjust predictors, and all models included cross-validated confound regression using three noxious-evoked response imaging confounds to adjust responses. Prediction performance was assessed using sums-of-squares formulation of the coefficient of determination (R^2), and each univariate model performance was qualitatively compared to each other and to the prediction performance of the three multivariate prediction models (Table 2 in main text). The results are displayed in Supplementary Figure 4b.

Similar to our multivariate prediction model results (Figure 4) and univariate correlation results (Supplementary Figure 4a), individual clinical variables and resting-state imaging confounds were not predictive of neonates' noxious-evoked response amplitudes (Supplementary Figure 4b). Of the nine resting-state networks, only the SMN exhibit notable predictive value achieving a prediction performance ($R^2 = 0.60$) comparable to our multivariate resting-state prediction model performance ($R^2 = 0.62$) (Supplementary Figure 4b). Thus, while eight of nine resting-state networks were positively correlated with noxious-evoked response amplitudes, notable network-to-network variability in positive association strength existed, and the most robustly associated and predictive network (SMN) was a network with high functional relevance to our noxious stimulation paradigm.

These results suggest that global network correlation polarities may reflect global brain maturity effects[4,5], while local network-to-network variability in correlation strengths may reflect local maturational asynchronies[6,7]. Thus in order to understand neonatal noxious-evoked response amplitudes, both global characteristics not specific to neural processing of noxious stimulation (e.g. maturational state) and local nociception-related characteristics (e.g. resting-state activity of functionally relevant networks) are needed. While we did not find maturity-related variables such as age and brain volume to have much predictive value in this study, it is likely these variables would increase their predictive value when considering a wider age range, so we do not conclude that age and brain volume are irrelevant to understanding neonates' noxious-evoked response amplitudes.

Cross-dataset similarities in functional and structural features

Our structure-function association analyses assessed the relationship between neonates' noxious-evoked response amplitudes and three white matter microstructural properties: mean diffusivity (MD), fractional anisotropy (FA), and mean kurtosis (MK). For these analyses, we adopted a two-part two-dataset approach involving exploration of a range of structure-function associations in the dHCP dataset (n=215) followed by confirmation of findings in our noxious stimulation paradigm dataset (n=17). In the noxious stimulation paradigm dataset, measured noxious-evoked response amplitudes are available. In the dHCP dataset, noxious-evoked response data are not available, so noxious-evoked response amplitudes are predicted per neonate from their resting-state data using our resting-state prediction model.

The two datasets were collected on different scanners using different diffusion and functional scan protocols – see Table 3 in main text for cross-dataset comparison of key acquisition parameters. These acquisition differences can introduce undesirable variability in the data that could cause issues in identification of replicable structure-function associations between datasets. Additionally, the reliance on predicted noxious-evoked response amplitudes in the dHCP dataset will contribute further variability due to unavoidable limitations in the accuracy of the resting-state prediction model. Here we qualitatively compared the distributions of the functional and structural features to identify any major inconsistencies between datasets. To calculate distributions for the three diffusion parameters, an average parameter value was calculated for each of the 16 white matter tracts per neonate. The results are displayed in Supplementary Figure 5.

Despite the differences in functional acquisition protocols and the reliance on predicted noxious-evoked response amplitudes in the dHCP dataset, the frequency distributions of noxious-evoked response amplitudes were very well aligned between the two datasets (Supplementary Figure 5, left). For each diffusion parameter, there was reasonably high consistency in frequency distributions between datasets, with greatest alignment visible for MD. Importantly, it was the structure-function associations that we attempted to replicate between datasets, so it is within-dataset relations between MRI modalities that is of primary interest. The minor cross-dataset variability in these functional and structural feature distributions (Supplementary Figure 5) is likely to have minimal influence on subject-to-subject variability within a dataset, and is thus unlikely to detrimentally impact comparisons of structure-function associations between datasets.

The close correspondence of both the functional and structural features (Supplementary Figure 5) and the structure-function associations between datasets (Figure 6 and Supplementary Figures 6-7) suggests that undesirable between-dataset inconsistencies has, at most, minor effects, and our resting-state prediction model generalises well to the age-matched dHCP data.

Structure-function associations: global effects, non-uniformity, and nociception-relevance

In our structure-function analysis, we defined 16 bilateral white matter tracts and assessed cross-subject variation in these tracts for three dMRI parameters: mean diffusivity (MD), fractional anisotropy (FA), and mean kurtosis (MK). We explored and identified nociception-related structure-function associations in the dHCP dataset by correlating the neonates' predicted noxious-evoked response amplitudes with these 48 dMRI features (16 tracts x 3 parameters). The results of this

exploratory analysis are presented in Figure 5 of the main text. We note two observations. First, there are global effects for both MD and FA: in all 16 tracts, there are negative associations between noxious-evoked response amplitudes and MD and positive associations between noxious-evoked response amplitudes and FA. Second, there is a local effect where statistically significant correlations were observed for the MD of five tracts, and these five tracts have high functional relevance to our noxious stimulation paradigm – see Discussion in main text. Furthermore, of these five tracts, the superior thalamic radiations and the corticospinal tracts form core structural connectivity for somatosensory and motor functions. This specific subset of functionally relevant tracts mirrors our identification of the resting-state SMN activity as most robustly associated with neonates' noxious-evoked response amplitudes (Supplementary Figure 4b). Thus, analogous to our resting-state functional coupling results above, our structure-function association results suggest both global and local effects are important features of the data. Here, we examine these effects further to gain additional insight into our structure-function association results.

To test for global structure-function effects in our noxious stimulation paradigm dataset, the Pearson correlation coefficients for each tract and diffusion parameter were generated using matched analysis to that in the dHCP dataset. The correlation results for both datasets are displayed together in Supplementary Figure 6a. As in the dHCP dataset, the noxious stimulation paradigm dataset exhibited global effects for both MD and FA: all 16 tracts exhibited negative correlations with MD, and 15 of 16 tracts exhibited positive associations with FA (the medial lemniscus exhibited a negative association). We attribute these global effects to brain maturity such that larger noxious-evoked response amplitudes and white matter tracts with smaller MD values and larger FA values are associated with a more functionally and structurally mature brain (see Discussion in main text).

Within both datasets, the structure-function association strengths are not uniform, but vary widely from tract to tract (Supplementary Figure 6a). In the dHCP dataset, the five statistically significant MD associations had the largest correlation values and were nociception-relevant. To test if this tract-to-tract variability in structure-function association strength was consistent between datasets, we correlated the association strength of individual tracts (each dataset's 16 Pearson r-values) between datasets for each diffusion parameter (Supplementary Figure 6b). The spatial autocorrelation between tracts means the observations within datasets are not independent, so we report effect sizes only and omit tests for statistical significance. Due to the presence of extreme values (e.g. medial lemniscus in FA), we exclude outliers from these assessments, where an outlier is defined as an observation with a Cook's Distance $D>1$[8] (Supplementary Figure 6b, yellow dots). Finally, we square the Pearson correlation to provide an estimate of shared variance (coefficient of variation). There was no meaningful similarity between datasets in tract-to-tract variability in association strength for FA (r^2=0.0044) or MK (r^2=0.019), suggesting the global positive associations for FA lack tract specificity. In contrast, there is a strong similarity between datasets for MD (r=0.62, r^2=0.38), suggesting the global negative association for MD exhibits a degree of tract specificity i.e. the tracts with strongest associations between MD and noxious-evoked response amplitudes are relatively consistent between datasets.

Lastly, we examined the relative functional importance to noxious-evoked response amplitudes of the MD of the five white matter tracts identified in the dHCP dataset exploratory analysis. The five tracts were the superior and anterior thalamic radiations, corticospinal tract, uncinate fasciculus, and forceps minor (Supplementary Figure 6a, red box). As described in the main text, in our noxious stimulation paradigm dataset, the first principal component of MD across these five tracts (MD PC1) explained over 20% of the variance in noxious-evoked response amplitudes (Figure 6b; r^2=0.21). We compared the r^2 value for this specific 5-tract combination against every possible combination of the 16 tracts (i.e. MD PC1 r^2 values for all unique combinations) (Supplementary Figure 7). This distribution of MD PC1 r^2 values had a median of r^2=0.17 and a robust range (1st and 99th percentiles) of [0.08, 0.24]. The specific 5-tract MD PC1 r^2=0.21 featured in the 92.3th percentile. Additionally, variance explained by the MD PC1 across all 16 tracts lay close to the median with a r^2=0.17 at the 55.8th percentile. Thus, in the noxious stimulation paradigm dataset, like the dHCP dataset, the MD of this specific subset of five white matter tracts exhibited high explanatory value for inter-individual variability in neonates' noxious-evoked response amplitudes and outperformed the global MD metric assessed across all tracts which does not take into account the tract-to-tract variability in functional relevance.

These results suggest that global correlation polarities of MD and FA may reflect global brain maturity effects[9], while local tract-to-tract variability in MD correlation strengths may reflect local maturational asynchronies[7,10,11]. Thus, analogous to our resting-state functional coupling results above, in order to understand neonatal noxious-evoked response amplitudes, both global characteristics not specific to neural processing of noxious stimulation (e.g. maturational state) and local nociception-related characteristics (e.g. microstructure of functionally relevant white matter tracts) are needed. Future work investigating the microstructural basis of these global and local structure-function association effects would be a valuable route of enquiry, and would likely benefit from complementary insights from biophysical models such as NODDI[12].

CSF and white matter regions-of-interest definition

When associating noxious-evoked response amplitudes with both resting-state fMRI features and white matter dMRI features, the functional data were adjusted for several imaging confounds. The noxious-evoked response amplitudes were adjusted for three noxious-evoked response imaging confounds (mean head motion, stimulus-correlated head motion, CSF amplitude), and the resting-state network amplitudes were adjusted for three resting-state imaging confounds (mean head motion, CSF amplitude, and white matter amplitude). The extraction of CSF and white matter amplitudes from the functional data required CSF and white matter ROI (region-of-interest) masks.

Due to the small size of the neonatal brain, partial volume contamination is problematic. The CSF and white matter masks used in our analyses were conservative to minimize grey matter contamination, and were defined using the dHCP neonatal template atlas[13]. The CSF ROI (Supplementary Figure 8 blue) is restricted predominantly to the fluid surrounding the brainstem and cerebellum and is a single region crossing the midline. Ventricular CSF was excluded from this ROI due to the small size of neonatal ventricles. The white matter ROI (Supplementary Figure 8 red) is restricted to the largest white matter regions in the cerebrum, consisting of a left and right half

tightly localized to the centre of white matter regions. In the resting-state data, these ROIs were used to extract mean timeseries for both CSF and white matter. The amplitudes of these timeseries were quantified as the timeseries MAD values. In the noxious-evoked response data, the CSF ROI was used to extract the mean regression parameter from neonates' noxious-evoked response maps, which constituted the noxious-evoked response CSF amplitude.

Supplementary References

1. Woolrich, M. W., Behrens, T. E. J., Beckmann, C. F. & Smith, S. M. Mixture models with adaptive spatial regularization for segmentation with an application to FMRI data. *IEEE Trans Med Imaging* **24**, 1–11 (2005).
2. Winkler, A. M., Ridgway, G. R., Webster, M. A., Smith, S. M. & Nichols, T. E. Permutation inference for the general linear model. *Neuroimage* **92**, 381–397 (2014).
3. Spisak, T. *et al.* Pain-free resting-state functional brain connectivity predicts individual pain sensitivity. *Nature Communications* **11**, 187 (2020).
4. Fitzgibbon, S. P. *et al.* The developing Human Connectome Project (dHCP) automated resting-state functional processing framework for newborn infants. *bioRxiv* 766030 (2019) doi:10.1101/766030.
5. Smyser, C. D. *et al.* Longitudinal analysis of neural network development in preterm infants. *Cereb. Cortex* **20**, 2852–2862 (2010).
6. Cao, M. *et al.* Early Development of Functional Network Segregation Revealed by Connectomic Analysis of the Preterm Human Brain. *Cereb. Cortex* **27**, 1949–1963 (2017).
7. Ouyang, M., Dubois, J., Yu, Q., Mukherjee, P. & Huang, H. Delineation of early brain development from fetuses to infants with diffusion MRI and beyond. *Neuroimage* **185**, 836–850 (2019).
8. Cook, R. D. & Weisberg, S. *Residuals and Influence in Regression*. (New York: Chapman and Hall, 1982).
9. Dubois, J. *et al.* The early development of brain white matter: a review of imaging studies in fetuses, newborns and infants. *Neuroscience* **276**, 48–71 (2014).
10. Dubois, J. & Dehaene-Lambertz, G. Fetal and postnatal development of the cortex: MRI and genetics. in *Brain Mapping: An Encyclopedic Reference* vol. 2 (Elsevier, 2015).
11. Dubois, J. *et al.* Asynchrony of the early maturation of white matter bundles in healthy infants: quantitative landmarks revealed noninvasively by diffusion tensor imaging. *Hum Brain Mapp* **29**, 14–27 (2008).
12. Zhang, H., Schneider, T., Wheeler-Kingshott, C. A. & Alexander, D. C. NODDI: practical in vivo neurite orientation dispersion and density imaging of the human brain. *Neuroimage* **61**, 1000–1016 (2012).
13. Schuh, A. *et al.* Unbiased construction of a temporally consistent morphological atlas of neonatal brain development. *bioRxiv* 251512 (2018) doi:10.1101/251512.
14. Fitzgibbon, S. *et al.* The developing Human Connectome Project automated functional processing framework for neonates. *OHBM* (2018).

Supplementary figures

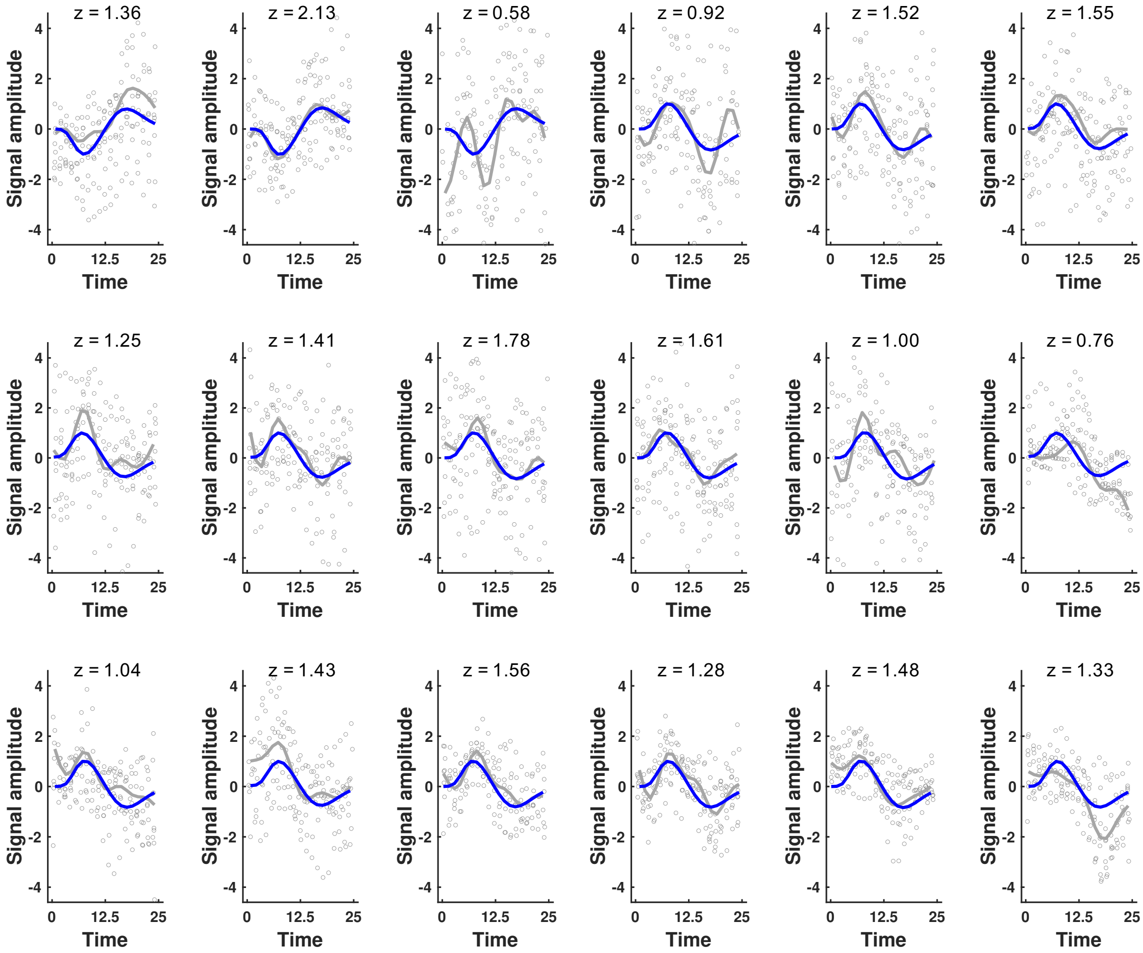

Supplementary Figure 1: Noxious-evoked response HRF fit. *The plots are displayed in identical order to Figure 1 (main text). For all plots, the x-axis (time in seconds) runs from 0 to 25, where t = 0 s is the point of stimulus delivery, and the minimum inter-stimulus interval is 25 s. The y-axis is arbitrarily scaled to blue curve (HRF fit) peak equals one. Grey circles are individual timepoints from all 10 trials, the grey line is the trial average timeseries, and the blue line is the HRF fit to the data. Above each plot is the z-score quantifying the goodness-of-fit between the HRF (blue line) and the data (grey line). Source data are provided as a Source Data file.*

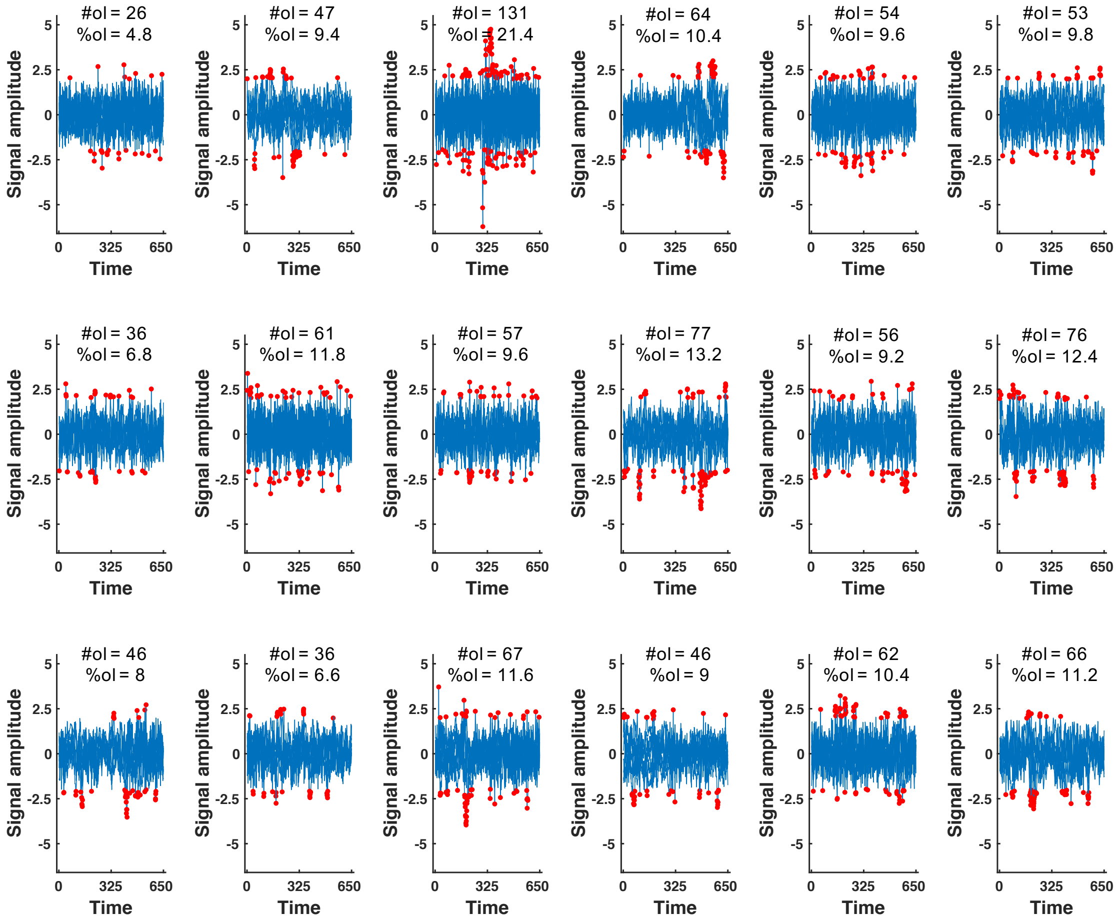

Supplementary Figure 2: Resting-state network timeseries and outliers. *The plots are displayed in identical order to Figure 1 in the main text. In each plot, the timeseries of all nine resting-state networks are plotted superimposed (blue), with the x-axis representing time in seconds (500 volumes = 650 s in total). The y-axis represents the network timeseries signal amplitudes. Each individual network timeseries has been independently zero-centred around its median and scaled to have unit interquartile range, resulting in all outliers, highlighted in red, having values greater than* ± 2*. Above each plot is the total number of outlier timepoints across all nine networks (#ol) and the percentage of unique timepoints labelled an outlier in at least one network timeseries (%ol). Source data are provided as a Source Data file.*

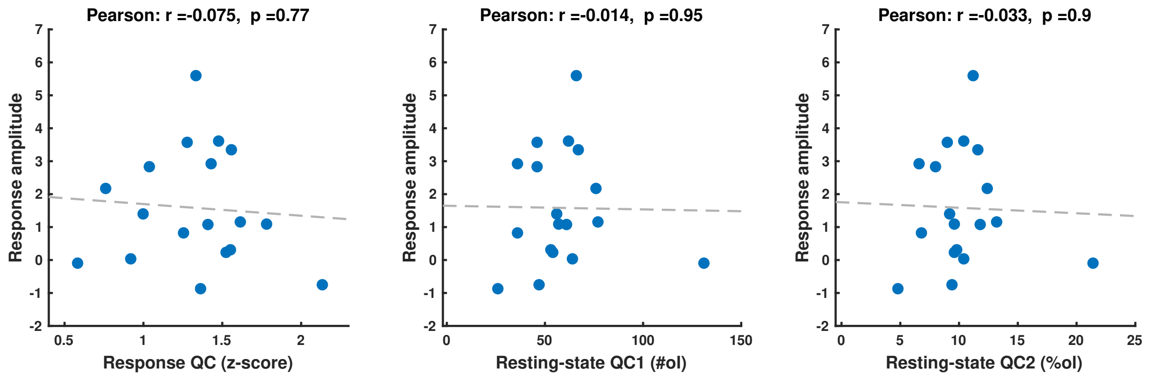

Supplementary Figure 3: Noxious-evoked response amplitudes vs fMRI data timeseries quality. *For all plots, the dashed grey line is the least squares line, and the Pearson correlation coefficient (r) and the associated two-sided p-value (p) are displayed overhead (n=18). Left: The correlation between noxious-evoked response amplitude (y-axis) and noxious-evoked response HRF goodness-of-fit (z-score values in Supplementary Figure 1). Middle: The correlation between noxious-evoked response amplitude and resting-state network timeseries total outlier timepoint count (#ol values in Supplementary Figure 2). Right: The correlation between noxious-evoked response HRF goodness-of-fit and resting-state network timeseries unique outlier timepoint percentage (%ol values in Supplementary Figure 2). Abbreviations: QC = quality control; #ol = number of outliers; %ol = percentage of outliers . Source data are provided as a Source Data file.*

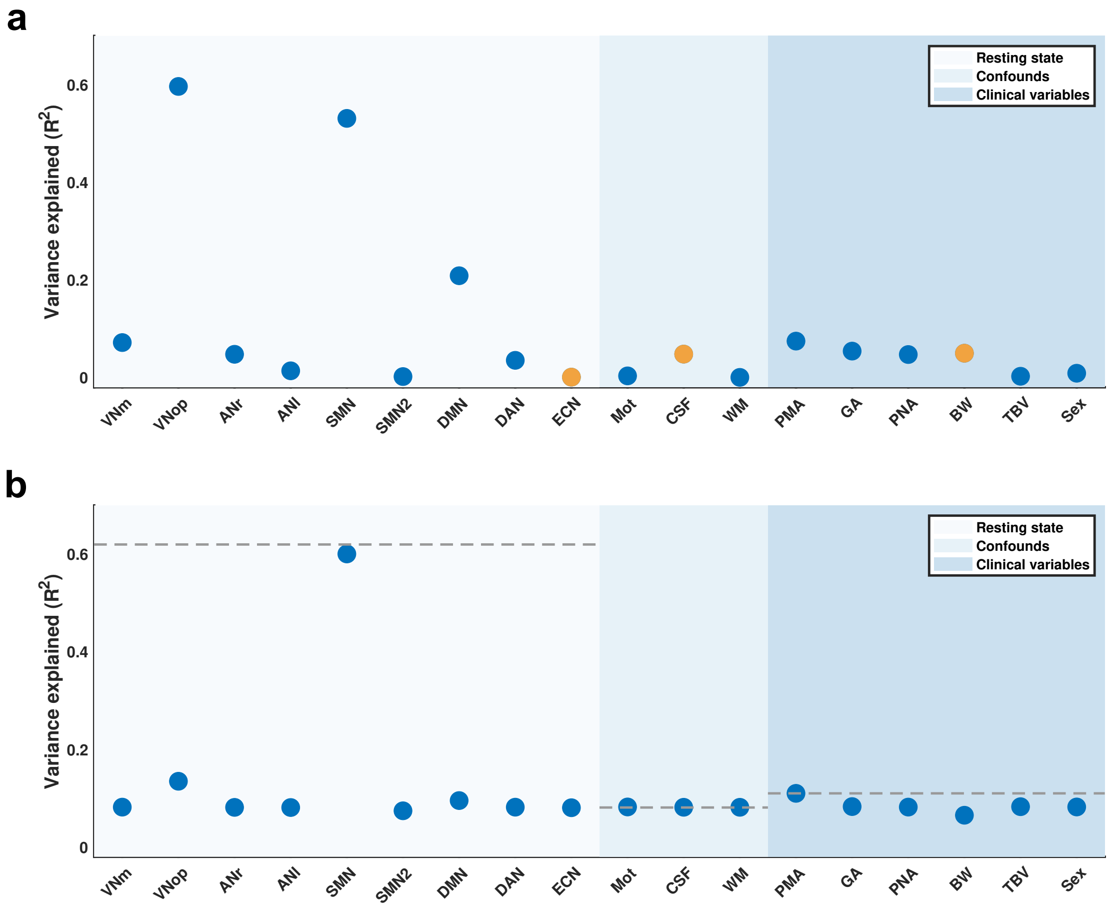

Supplementary Figure 4: Resting-state coupling: global effects, non-uniformity, and nociception-relevance. ***a:*** *Univariate correlations between neonates' noxious-evoked response amplitudes and each of the 18 predictors in our three predictor categories: resting-state networks (resting-state), resting-state imaging confounds (confounds), and clinical variables. Correlation strengths are presented as proportion of variance explained (Pearson R^2). Positive correlations are in blue and negative in yellow. Of the 18 Pearson correlation tests, only VNop and SMN were statistically significant. Of the nine resting-state networks, eight exhibited positive correlations.* ***b:*** *Univariate predictions between neonates' noxious-evoked response amplitudes and each of the 18 predictors. Prediction strengths are presented as proportion of variance explained (sums-of-squares R^2). The dashed grey lines indicate the prediction performance of each of the three multivariate prediction models for reference. Of all 18 predictors, only the SMN demonstrates meaningful predictive value, closely matching the overall performance of the multivariate resting-state prediction model. Abbreviations: VNm = medial visual network; VNop = occipital pole visual network; ANr = right auditory network; ANl = left auditory network; SMN = somatomotor network; DMN = default mode network; DAN = dorsal attention network; ECN = executive control network; Mot = head motion; CSF = cerebrospinal fluid signal amplitude; WM = white matter signal amplitude; PMA = postmenstrual age; GA = gestational age; PNA = postnatal age; BW = birth weight; TBV = total brain volume. Source data are provided as a Source Data file.*

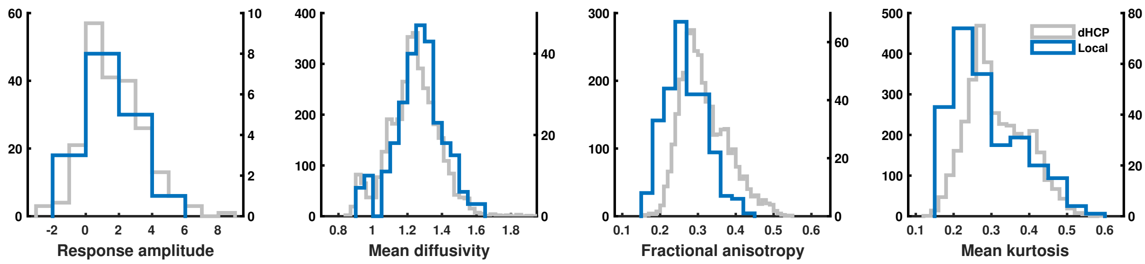

Supplementary Figure 5: Cross-dataset comparison of functional and structural features. *For each frequency histogram, the dHCP dataset (n=215) distribution is in grey with the y-axis scale on the left, and the local noxious stimulation paradigm dataset (n=18 for response amplitude histogram; n=17 for microstructure histograms) is in blue with the y-axis scale on the right. For the response amplitudes, the noxious stimulation paradigm dataset values are observed amplitudes, and the dHCP dataset values are predicted amplitudes. For the three white matter microstructural features, each infant contributed 16 values per parameter, an average parameter value for each of the 16 white matter tracts studied. Source data are provided as a Source Data file.*

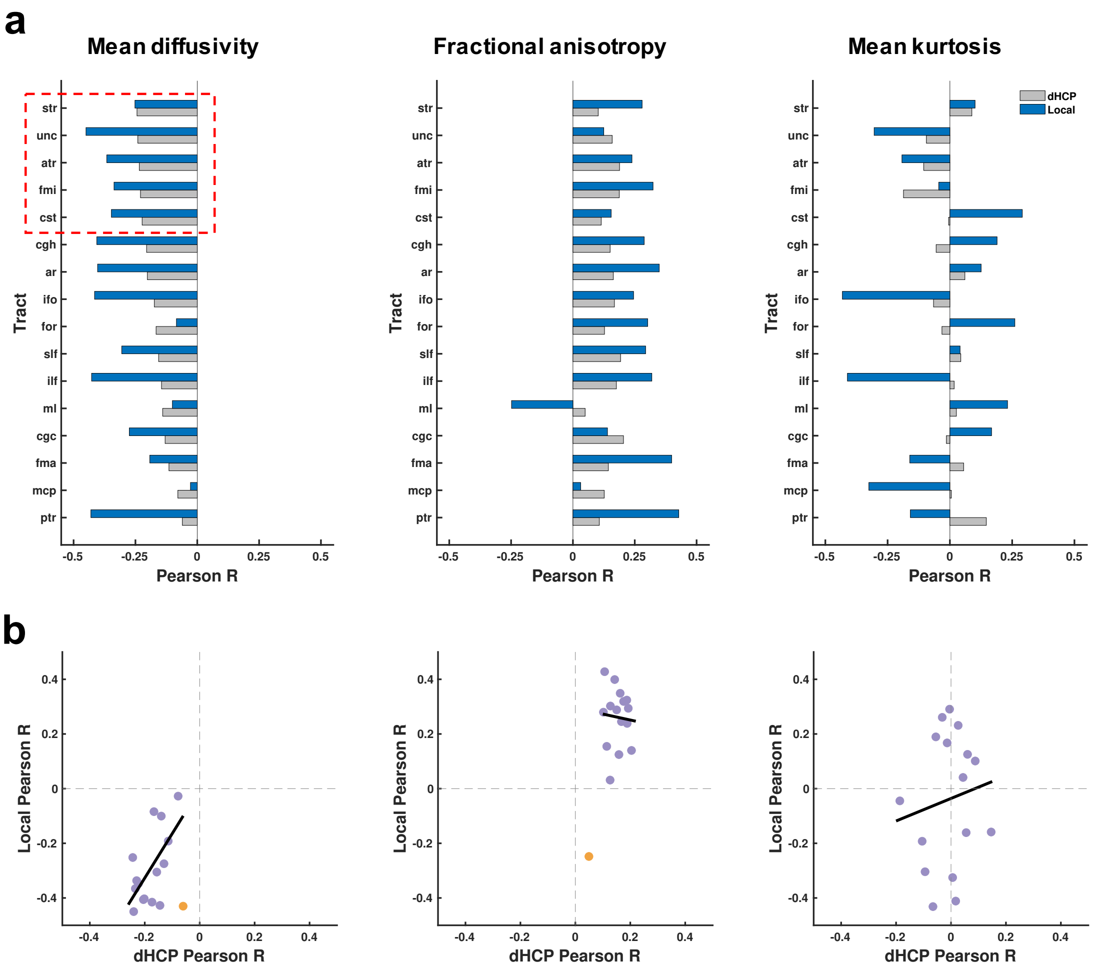

Supplementary Figure 6: Cross-dataset similarities in structure-function associations. a. *Bar plots displaying the Pearson correlation coefficients between noxious-evoked response amplitudes and microstructural features for each white matter tract. For each tract, the coefficients for both the dHCP dataset (grey) and noxious stimulation paradigm (local) dataset (blue) are juxtaposed. Tracts are ordered consistently according to the dHCP MD correlation coefficients: from negative (top) to positive (bottom). The red box indicates the five dHCP correlation coefficients identified as statistically significant in our exploratory arm analyses (Figure 5).* ***b.*** *Correlation of association strength for each tract across datasets. For each scatter plot, the dots indicate individual tracts with outliers highlighted in yellow. The black lines are the least squares fit lines, excluding outliers. The plots are divided into equal quadrants to illustrate polarity of the associations. Tracts with consistent polarities between datasets fall in the lower left and upper right quadrants. The global negative MD associations are visible as localisation of points to the lower left quadrant, and the global positive FA association are visible as localisation to the upper right quadrant. Abbreviations: ar = acoustic radiation; atr = anterior thalamic radiation; cgc = cingulate gyrus part of the cingulum; cgh = parahippocampal part of the cingulum; cst = corticospinal tract; fma = forceps major; fmi = forceps minor; for = fornix; ifo = inferior fronto-occipital fasciculus; ilf = inferior longitudinal fasciculus; mcp = middle cerebellar peduncle; ml = medial lemniscus; ptr = posterior thalamic radiation; slf = superior longitudinal fasciculus; str = superior thalamic radiation; unc = uncinate fasciculus. Source data are provided as a Source Data file.*

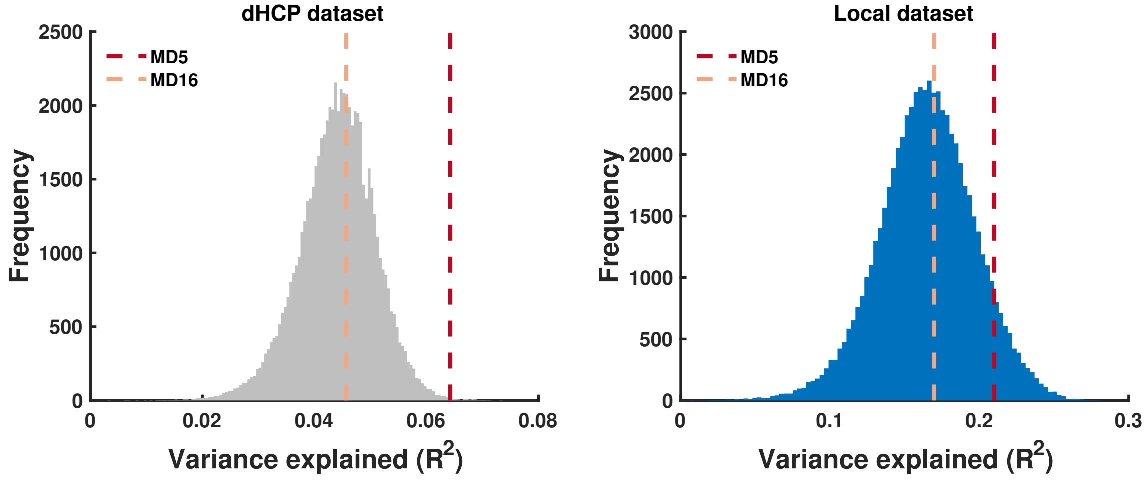

Supplementary Figure 7: Explanatory value of functionally relevant tracts' MD relative to whole brain MD. *For both histograms, the explanatory variable is the first principal component across the MD of white matter tracts (MD PC1). Pearson correlations between MD PC1 and noxious-evoked response amplitudes are squared to calculate the proportion of variance explained (x-axis). The distributions are constructed from all possible unique combination of the 16 tracts. The red lines (MD5) are the results for the specific combination of five nociception-relevant tracts identified in our dHCP dataset exploratory analyses (Supplementary Figure 6a, red box). The yellow lines (MD16) are the global MD PC1 results calculated across all 16 tracts. Left: These results from the dHCP dataset are biased due to the circularity in feature selection approach for MD5, but this plot demonstrates the effect-of-interest: the specific MD feature calculated from a subset of functionally relevant tracts has greater explanatory value than the global MD feature which ignores variability in tract relevance. Right: The specific 5-tract MD feature (MD5) exhibits greater explanatory value than the global 16-tract MD feature (MD16). Distribution median r^2=0.17; distribution robust range (1st and 99th percentiles) = [0.08, 0.24]; MD5 r^2=0.21 (92.3^{th} percentile); MD16 r^2=0.17 (55.8^{th} percentile). Source data are provided as a Source Data file.*

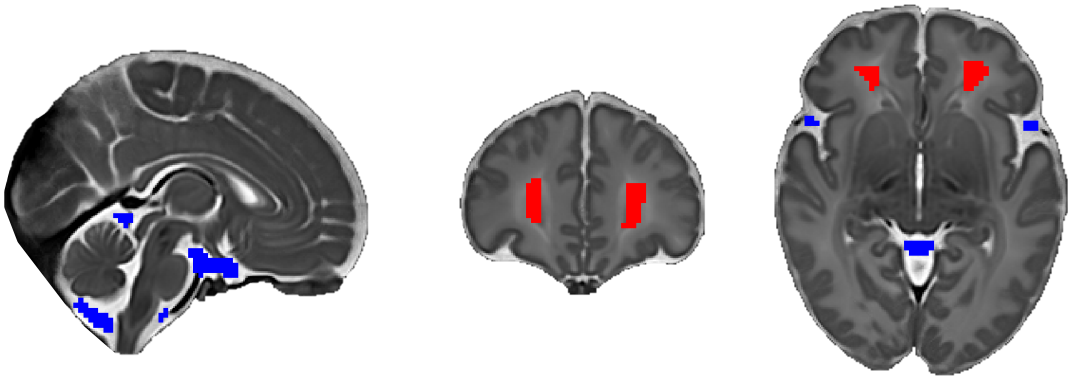

Supplementary Figure 8: CSF and white matter ROIs. *These anatomically defined regions were used to extract CSF and white matter signal features from both resting-state and noxious-evoked activities for confound signal assessment and confound adjustment. The CSF ROI (blue) is restricted predominantly to the fluid surrounding the brainstem and cerebellum. The white matter ROI (red) is restricted to the largest white matter regions in the cerebrum tightly localized to the centre of white matter regions. Abbreviations: CSF = cerebrospinal fluid; ROI = region of interest. Source data are provided as a Source Data file.*

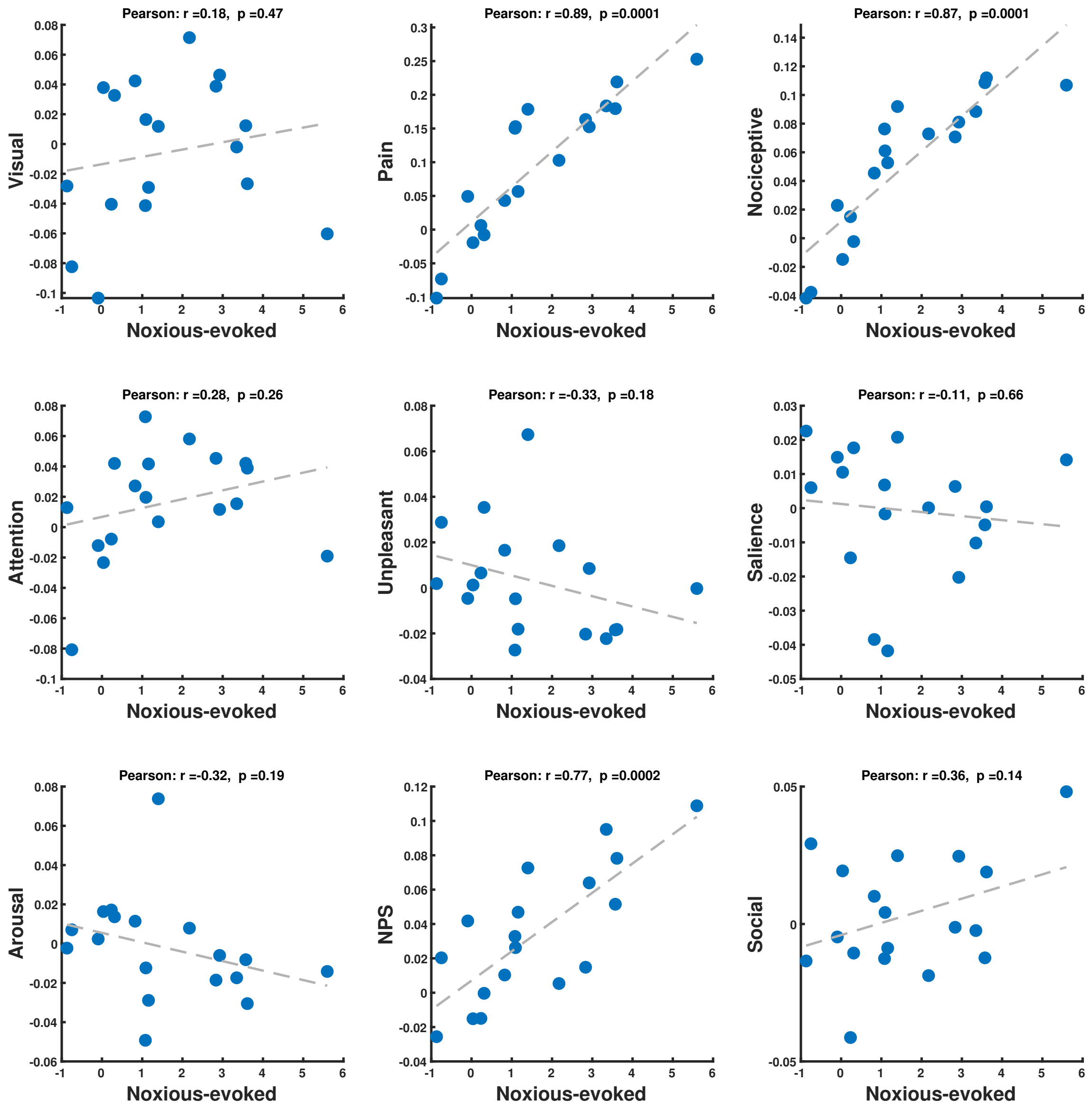

Supplementary Figure 9: Associations between inter-individual variability in noxious-evoked response amplitudes and functional template correspondence. *In all plots, each blue dot is a single subject (n=18), the dashed grey line is the least-squares fit, the x-axis is the overall noxious-evoked response amplitude (Figure 1 scalar values), and the y-axis is the correspondence (Pearson correlation coefficient) between each neonate's noxious-evoked response map (Figure 1 heat maps) and an adult functional template. Associations between noxious-evoked response amplitudes and adult functional template correspondence was assessed using two-tailed Pearson correlation tests. For all plots, the Pearson correlation coefficient (r) and the associated two-sided uncorrected p-value (p) are displayed overhead. Statistical results for the nine adult templates are presented in Table 1. Of the nine adult templates assessed, only the NPS and Neurosynth Pain and Nociceptive templates had statistically significant associations (after correction for multiple testing), such that neonates with larger noxious-evoked response amplitude had noxious-evoked response maps exhibiting closer correspondence with adult signatures of pain and nociception. Source data are provided as a Source Data file.*

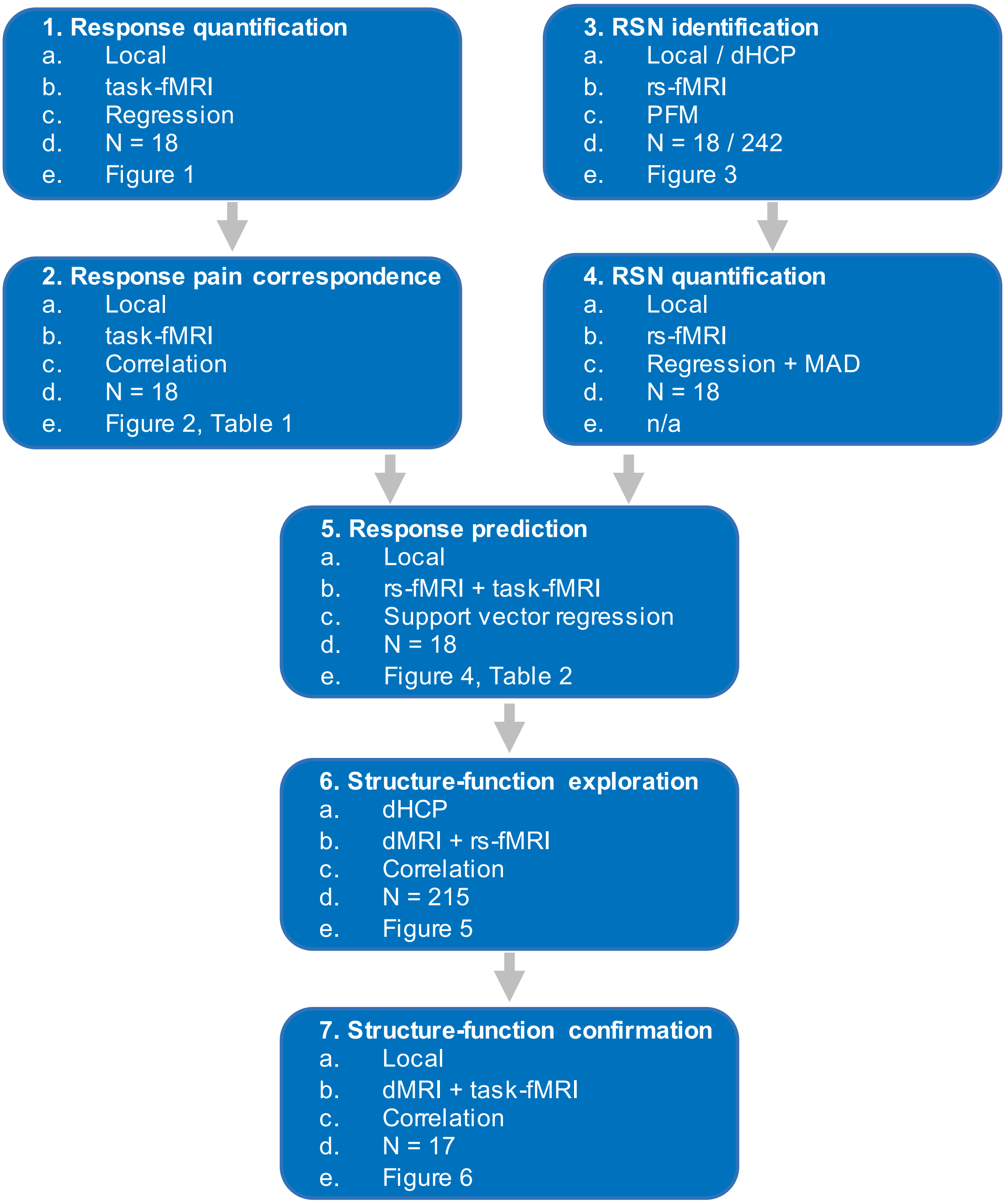

Supplementary Figure 10: Study workflow. *Sequence of key steps (1-7), each step summarised using five details (a-e), for the current study. Note in step 3, the dHCP dataset (n=242) analysis and results were generated prior to and independent of the current study in a previous dHCP research output[14]. Abbreviations: a = dataset; b = imaging modality; c = key analysis method; d = sample size; e = key results figures and tables; dHCP = developing human connectome project; fMRI = functional MRI; dMRI = diffusion MRI; rs-fMRI = resting-state fMRI; RSN = resting-state network; MAD = median absolute deviation; PFM = probabilistic functional mode analysis.*
