# Supplementary material for: Functional and diffusion MRI reveal the neurophysiological basis of neonates’ noxious-stimulus evoked brain activity
Source: Nat Commun. 2021 May 12;12:2744. doi: 10.1038/s41467-021-22960-0 (PMC8115252; doi:10.1038/s41467-021-22960-0)
Supplement: Supplementary file 3 — Reporting Summary [file 41467_2021_22960_MOESM3_ESM.pdf]

nature research

Corresponding author(s): Rebeccah Slater

Last updated by author(s): Feb 17, 2021

Reporting Summary

Nature Research wishes to improve the reproducibility of the work that we publish. This form provides structure for consistency and transparency in reporting. For further information on Nature Research policies, seeAuthors & Referees and theEditorial Policy Checklist .

Statistics

For all statistical analyses, confirm that the following items are present in the figure legend, table legend, main text, or Methods section.

n/a	Confirmed	
☐	☒	The exact sample size (*n*) for each experimental group/condition, given as a discrete number and unit of measurement
☐	☒	A statement on whether measurements were taken from distinct samples or whether the same sample was measured repeatedly
☐	☒	The statistical test(s) used AND whether they are one- or two-sided<br>*Only common tests should be described solely by name; describe more complex techniques in the Methods section.*
☐	☒	A description of all covariates tested
☐	☒	A description of any assumptions or corrections, such as tests of normality and adjustment for multiple comparisons
☐	☒	A full description of the statistical parameters including central tendency (e.g. means) or other basic estimates (e.g. regression coefficient) AND variation (e.g. standard deviation) or associated estimates of uncertainty (e.g. confidence intervals)
☐	☒	For null hypothesis testing, the test statistic (e.g. *F*, *t*, *r*) with confidence intervals, effect sizes, degrees of freedom and *P* value noted<br>*Give P values as exact values whenever suitable.*
☒	☐	For Bayesian analysis, information on the choice of priors and Markov chain Monte Carlo settings
☒	☐	For hierarchical and complex designs, identification of the appropriate level for tests and full reporting of outcomes
☐	☒	Estimates of effect sizes (e.g. Cohen's *d*, Pearson's *r*), indicating how they were calculated

Our web collection on statistics for biologists contains articles on many of the points above.

Software and code

Policy information about availability of computer code

Data collection	To collect the noxious-response fMRI data, the stimulus applications were time-locked using the Presentation software package from Neurobehavioural Systems software v20.1 (https://www.neurobs.com/)
Data analysis	Prediction modelling was performed using Python v3.7.4 and Scikit-learn v0.21.3<br><br>Cross-validated confound regression was performed using MVCA (https://github.com/lukassnoek/MVCA)<br><br>MRI data analysis was performed using FSL v6.0.3, MIRTK Draw-EM neonatal pipeline v1.1, dHCP fMRI pipeline v0.5.3, dHCP dMRI pipeline v0.0.2<br><br>Some basic calculations and figure plotting was performed using MATLAB v9.7.0

For manuscripts utilizing custom algorithms or software that are central to the research but not yet described in published literature, software must be made available to editors/reviewers. We strongly encourage code deposition in a community repository (e.g. GitHub). See the Nature Research guidelines for submitting code & software for further information.

Data

Policy information about availability of data

All manuscripts must include a data availability statement. This statement should provide the following information, where applicable:

- Accession codes, unique identifiers, or web links for publicly available datasets
- A list of figures that have associated raw data
- A description of any restrictions on data availability

Source data are provided with this paper. The noxious stimulation paradigm data that support the findings of this study are available from the corresponding author upon reasonable request. The dHCP data (Second Data Release) that support the findings of this study are available online (http://www.developingconnectome.org/

second-data-release).

Field-specific reporting

Please select the one below that is the best fit for your research. If you are not sure, read the appropriate sections before making your selection.

☒ Life sciences ☐ Behavioural & social sciences ☐ Ecological, evolutionary & environmental sciences

For a reference copy of the document with all sections, see nature.com/documents/nr-reporting-summary-flat.pdf

Life sciences study design

All studies must disclose on these points even when the disclosure is negative.

Sample size	Noxious stimulation paradigm dataset: 21 infants' MRI data were collected within the recruitment period for this study. Three subjects' data did not meet inclusion criteria (outlined in paper), so the sample size was n=18.<br><br>dHCP dataset: subjects were selected to match the prediction model training set i.e. to match the noxious stimulation paradigm dataset. The sample size was determined by the number of subjects matching our inclusion criteria that were available in the Second Data Release (n=215).<br><br>The sample sizes were deemed sufficient due to the successful application of the model to identify consistent findings across the exploration and confirmation analysis arms, as outlined in the manuscript.
Data exclusions	Noxious stimulation paradigm dataset:<br>Subjects were excluded from analysis if (i) either scan session (noxious-response or resting-state) was not fully completed, in order to remove inter-subject variability in data quality related to scan length, and (ii) the vertex of the cerebral cortex left the scan field of view for more than 5% of the scan session, in order to ensure reliable data in this functionally relevant brain region.<br><br>dHCP dataset:<br>Subjects were selected to match our noxious stimulation paradigm sample. Neonates were included in our dHCP dataset sample if data were of reasonable quality and neonates were age-matched to our noxious stimulation paradigm dataset. The three data quality criteria were: (i) both fMRI and dMRI data had to pass basic dHCP QC pipelines, (ii) both scan sessions had to have completed fully, and (iii) the vertex of the cerebral cortex had to remain within the scan field of view for at least 95% of the scan session. The two age criteria were: (i) neonates had to have both GA and PMA between 36–42 weeks, and (ii) neonates had to have been scanned within the first 10 postnatal days.
Replication	The results are generated using standard available commands available in the software packages used: FSL, MATLAB, Scikit-learn. These commands were scripted, saved, and reviewed and run by two independent researchers (authors LB and MA) to ensure reproducibility. While we do not have an independent dataset to directly test replicability of our key prediction results, our exploratory-confirmatory analysis approach indirectly validates this result i.e. establishes indirect external validation. This is a key aspect of the paper, and the approach and rationale are detailed in the manuscript.
Randomization	Both datasets are single-sample, so no randomisation was needed. Covariates were controlled for by including them in the relevant models as nuisance variables. This is outlined in detail in the manuscript.
Blinding	There is no group allocation, so blinding was not required.

Reporting for specific materials, systems and methods

We require information from authors about some types of materials, experimental systems and methods used in many studies. Here, indicate whether each material, system or method listed is relevant to your study. If you are not sure if a list item applies to your research, read the appropriate section before selecting a response.

Materials & experimental systems

n/a	Involved in the study	
☒	☐	Antibodies
☒	☐	Eukaryotic cell lines
☒	☐	Palaeontology
☒	☐	Animals and other organisms
☐	☒	Human research participants
☒	☐	Clinical data

Methods

n/a	Involved in the study	
☒	☐	ChIP-seq
☒	☐	Flow cytometry
☐	☒	MRI-based neuroimaging

Human research participants

Policy information about studies involving human research participants

Population characteristics	Noxious stimulation paradigm dataset:<br>Postmenstrual age: mean = 38.7 weeks, standard deviation 1.7 weeks.<br>Sex: 10 males, 8 females<br><br>dHCP dataset:<br>Postmenstrual age: mean = 40.1 weeks, standard deviation 1.4 weeks.<br>Sex: 122 males, 93 females
Recruitment	We recruited healthy neonates from the postnatal ward at the John Radcliffe Hospital if they were inpatients on the postnatal ward that never required admission to the neonatal unit, had no history of congenital conditions or neurological problems, and were clinically stable at the time of study.<br><br>The need to transport neonates within the hospital from the postnatal ward to the imaging centre as part of the study limits recruitment to healthy neonates, and thus limits the focus to the normative neonatal population. As mentioned in the manuscript, this recruitment bias makes it unclear how well our observations will generalise to other populations, such as those born very prematurely.
Ethics oversight	NHS Research Ethics Committee (National Research Ethics Service)

Note that full information on the approval of the study protocol must also be provided in the manuscript.

Magnetic resonance imaging

Experimental design

Design type	Both resting-state design and task design (noxious stimulation paradigm) were used. The task design was event-related.
Design specifications	The task design included 10 trials per subject, 1 s trial duration, and a minimum of 25 s inter-trial interval.
Behavioral performance measures	As the subjects were neonates, there are no behavioural performance measures. Subject head motion was a subject behaviour that was quantified at the data analysis stage from the MRI data using standard image analysis tools, as described in the manuscript.

Acquisition

Imaging type(s)	Functional (resting-state and "task") and diffusion.
Field strength	3 T
Sequence & imaging parameters	Functional:<br>Pulse sequence = gradient echo<br>Readout = EPI<br>FOV = 180 mm<br>Matrix size = 90 x 90<br>Slice thickness = 2 mm<br>Orientation = axial slices<br>TE = 50 ms<br>TR = 1,300 ms<br>Flip angle = 70 degrees<br><br>Diffusion:<br>Pulse sequence = spin echo<br>Readout = EPI<br>FOV = 180 mm<br>Matrix size = 102 x 102<br>Slice thickness = 1.75<br>Orientation = axial slices<br>TE = 73 ms<br>TR = 2,900 ms<br>Flip angle = 90 degrees
Area of acquisition	Whole-brain

Diffusion MRI ☒ Used ☐ Not used

Parameters	# of directions = 143<br>b-values = 0, 500, 1000, 2000<br>multishell = yes<br>cardiac gating = no

Preprocessing

Preprocessing software	Functional:<br>dHCP fMRI preprocessing pipeline v0.5.3 with default parameters, which includes:<br>EDDY v9.1<br>FIX v1.06.15<br>Temporal filtering (high-pass) at 0.01 Hz for task data and 0.005 Hz for resting-state data<br>Spatial filtering (low-pass) with 3 mm FWHM<br><br>Diffusion:<br>dHCP dMRI preprocessing pipeline v0.0.2 with default parameters, which includes EDDY v9.1
Normalization	Native-to-structural:<br>Linear registration using 6-DOF rigid body alighment refined with BBR, using FSL's FLIRT. For functional data, alignment used single-band reference image (SBref) as intermediate.<br><br>Structural-to-standard:<br>Non-linear using ANTs's SyN
Normalization template	dHCP 40-week neonate T2 template (Schuh et al., 2018)
Noise and artifact removal	Functional:<br>Structured noise timeseries were identified using spatial ICA-based decomposition followed by manual identification of structured noise components.<br>Six motion parameter timeseries were defined by EDDY during preprocessing, which were expanded to the standard 24 motion parameter timeseries (including the quadratic and temporal derivatives).<br>The structured noise timeseries and motion parameter timeseries were simultaneously removed using FSL's FIX.
Volume censoring	None

Statistical modeling & inference

Model type and settings	Subject-level:<br>Mass univariate general linear modelling (GLM)<br>HRF model was the term infant double gamma function<br>Temporal autocorrelation was accounted for using FSL's default prewhitening (FILM prewhitening)<br><br>Group-level<br>Mass univariate general linear modelling (GLM)
Effect(s) tested	For the stimulus condition, the precise effect that was modelled was the voxelwise stimulus response amplitude relative to baseline, defined as the double gamma HRF scaling factor (regression coefficient).

Specify type of analysis: ☒ Whole brain ☐ ROI-based ☐ Both

Statistic type for inference<br>(See Eklund et al. 2016)	Subject level:<br>Subjects' statistical parametric maps were used unthresholded i.e. there was no voxel-wise or cluster-wise thresholding, and statistical inferences were made on features extracted from unthresholded maps.<br><br>Group level:<br>Non-parametric cluster-based thresholding was used to threshold the group-average noxious-response map. The following details are taken directly from our text.<br>"To localise the noxious-evoked activity, we generated a thresholded group average activity map using group-level voxelwise GLM analysis in FSL's Randomise (Winkler et al., 2014). Statistical significance was assessed using permutation testing with 10,000 permutations, variance smoothing (6 mm FWHM kernel) due to limited degrees of freedom (Holmes et al., 1996), cluster-based thresholding with z=3.1 (p=0.001) cluster-defining threshold (Woo et al., 2014), and a FWER-corrected cluster p-value of p=0.05."
Correction	"To localise the noxious-evoked activity, we generated a thresholded group average activity map using group-level voxelwise GLM analysis in FSL's Randomise (Winkler et al., 2014). Statistical significance was assessed using permutation testing with 10,000 permutations, variance smoothing (6 mm FWHM kernel) due to limited degrees of freedom (Holmes et al., 1996), cluster-based thresholding with z=3.1 (p=0.001) cluster-defining threshold (Woo et al., 2014), and a FWER-corrected cluster p-value of p=0.05."

Models & analysis

n/a	Involved in the study
x	Functional and/or effective connectivity
x	Graph analysis
	x Multivariate modeling or predictive analysis

Multivariate modeling and predictive analysis	Features:<br>Clinical variables: six demographic details (includes ages, birth weight, brain volume, and sex)<br>Resting-state: Nine network amplitudes (networks identified using PFM analysis) and three imaging confounds (white matter amplitude, CSF amplitude, and mean head motion).<br>Noxious-response: One response amplitude and three imaging confounds (CSF amplitude, mean head motion, and stimulus-correlated head motion).<br><br>Independent variables:<br>Three groups of predictors: resting-state network amplitudes, resting-state imaging confounds, and clinical variables.<br><br>Dependent variable:<br>Noxious-response amplitude<br><br>Model:<br>Support vector regression (SVR)<br><br>Training:<br>Leave-one-out cross validation<br><br>Evaluation metrics:<br>Coefficient of determination (sums of squares formulation)<br>Root mean squared error<br>Spearman rank correlation coefficient
